# Fungal Endophytic Community and Diversity Associated with Desert Shrubs Driven by Plant Identity and Organ Differentiation in Extremely Arid Desert Ecosystem

**DOI:** 10.3390/jof7070578

**Published:** 2021-07-20

**Authors:** Yiling Zuo, Xia Li, Jingya Yang, Jiaqiang Liu, Lili Zhao, Xueli He

**Affiliations:** School of Life Sciences, Hebei University, Baoding 071002, China; zuoyiling@foxmail.com (Y.Z.); hbusummerli@hbu.edu.cn (X.L.); yangjingya196@foxmail.com (J.Y.); 18790522863@163.com (J.L.); zhaolili@hbu.edu.cn (L.Z.)

**Keywords:** mycobiota, community composition, plant identity, co-occurrences network, host–microbial interaction

## Abstract

Despite desert ecosystem being crucial to our understanding of natural geography, species evolution and global climate change, there is limited information on the dynamics of their composition and the diversity of endophytic fungi communities driven by plant identity and organ differentiation. Here, an extensive investigation of endophytic fungal microbiome in root, stem, and leaf organs associated with five xerophyte shrubs in an extremely arid desert, Northwest China, were examined. The fungal community dominated by *Dothideomycetes* and *Pleosporales*. Shrub species strongly drive the niche-based processes of endophytic fungi across the root, stem and leaf compartments. The diversity and composition of endophytic fungi in stem showed higher variability among plant species than leaf and root. The fungal communities in root libraries were more diverse and exhibited a remarkable differentiation of community composition. We further demonstrated the significant host preferences and tissue specificity of desert endophytic fungi, and unique specific taxa were also observed. The co-occurrence network revealed the coexistence of fungal endophytes in arid desert, and the root fungal network harbored the highest interspecies connectivity. Members of *Pleosporales* were the most common keystone species in the root fungal network. This is the first report of mycobiota in both plant species and organ differentiation in an extremely arid desert ecosystem.

## 1. Introduction

Xerophyte shrubs have strong drought resistance and usually grow and survive in environments with low water availability and nutrient deprivation [1]. Research on xerophyte plants is of particular interest due to their diverse strategies to survive in such harsh environments [2,3]. Special attention has been paid to the strategies consisting of associated microorganisms, such as fungal endophytes, that ameliorate plant growth and fitness under drought resistance [4,5]. Fungal microbiota colonizes their plant host to adapt themselves to the plant’s environment; hence, the symbionts have coevolved for millions of years [6]. The establishment of particular fungus–plant mutualistic relationships can confer thermotolerance, drought resistance, and a multitude of functional capabilities that enhance the survival, primary productivity and community structure of plants [7,8,9]. Considering the environmental filter of drought, fungal endophytes of xerophyte shrubs may be different and would bear a unique assemblage of drought tolerance that performs various ecological roles in protecting the host plant in arid desert [10]. Thus, elucidating the patterns of occurrence and ecological processes of the fungal microbiome in desert ecosystems is of great importance.

As a consequence of the reliance of endophytic fungi on the plant’s inner environment for growth, it is presumed that plant identity, therefore, plays a particularly decisive role in the fungal colonization of internal tissues [11]. Plants can recruit microbiota by releasing specific molecular signals and microorganisms capable of recognizing the signals could preferentially and successfully colonized the different plant organ niches [12,13]. Alternatively, plants exert selection pressure and a filtering effect via the immune system and the supply of specialized nutrients and habitat types [4,14]. In arid desert areas, different shrubs have evolved with differing morphological and physiological organs and generated heterogeneous resource-rich patches, which reveals the potential of the host species to induce the establishment of specific microbiomes [15,16,17,18]. However, how the host species shapes microbial assembly across different organ niches is still undocumented.

Host plants potentially represent distinct microhabitats, such as the soil rhizosphere and root, stem and leaf endospheres, which may host contrasting fungal communities by providing specific biotic and abiotic conditions for the resident microorganisms [4,19,20]. Most of the attention has been dedicated to the niche differentiation of microbes at the rhizospheric soil–root interface [15,21,22] in the leaf and root endospheres [23,24,25], and even in the different organs of plants [17,19,26,27]. Despite evidence of the preference of fungi to a specific host, studies have demonstrated that fungi, to a certain extent, exhibit relative tissue specificity [26,27,28,29]. Different ecological processes might contribute to a high degree of organ specificity for fungal endophytic communities [25]. In an arid ecosystem, the different physiological responses of exposed photosynthetic and belowground systems to environmental stress were suspected of producing the differentiation of fungal communities. Yet, we still know little about the endophytic fungal assembly between above- and belowground compartment niches, particularly for desert shrubs in extremely arid environments.

Multispecies assemblages usually do not live in isolation but instead form interactive relationships through mutualistic or competitive connections [30]. These interactive connections ultimately result in the construction of complex interspecies networks that maintain the structure of an ecological community and ecosystem functions [31]. Co-occurrence network analysis have been proven to be a useful tool for analyzing microbial relationships and have recently been used to study complex ecological systems, such as bacterial or fungal communities in various environments [32,33,34]. The characteristics of keystone species, member interactions and community organization can be predicted by network analysis to determine the importance of operational taxonomic units (OTUs) and their associations in the community [35,36]. Network analysis requires a tremendous amount of metadata to obtain strong statistical analysis, leading to trustable correlations; however, classical culture-dependent methods have limited ability to adequately describe microbial communities [17,37]. New high-throughput technologies have become available, showing a far greater sequencing capacity, which can provide the opportunity to explore communities with the computation method to reveal underlying species coexistence and assembly in the community [38].

The Anxi Extreme Arid Desert of the National Nature Reserve, located in Gansu Province, Northwest China, is the only natural reserve that focuses on the protection of extremely arid desert ecosystems and their associated biodiversity [39]. The native vegetation composed of xerophytic shrubs is highly adapted to their environment and has coevolved with drought-tolerant microorganisms for a long time. It is speculated that inter-organismal endophytes confer the ability of the host to tolerate drought and allow these plants to become pioneers in extremely arid desert [40,41]. This naturally arid desert system provides the optimal setting to elucidate the coevolution of the plant host–microbiome relationship. To the best of our knowledge, no reports on the co-occurrence patterns of the endophytic fungi associated with these prevalent plant species in extreme arid deserts are available.

Our previous investigations predominately focused on the spatial patterns of root-associated fungi, such as the arbuscular mycorrhizal fungi and dark septate endophytes that are symbiotic with ecologically important plants in arid desert [42,43,44,45,46]. In the present study, we examined the endophytic fungal microbiome and co-occurrence network across the root, stem, and leaf compartment niches associated with five endemic shrubs in an extremely arid desert, Northwest China. We aimed to understand how the communities of endophytic fungi varied across organ microhabitats within the plant host and among host species under identical environmental conditions. We addressed the following questions: (1) How variable are the fungal communities associated with different plants within the same area in the extremely arid desert? (2) Do the endosphere fungal communities vary among the different plant compartment niches? (3) How do the aboveground fungal communities relate to the belowground fungal communities? Understanding the host–microbe interactions of desert plants could provide the basis for the exploitation of the plant–fungi associations in manipulation of plants’ microbiome for ecological restoration.

## 2. Materials and Methods

### 2.1. Study Site and Sampling

The study was conducted in the Anxi Extreme Arid Desert of the National Nature Reserve in Gansu Province (40°06′−40°08′ N, 96°17′−96°36′ E), Northwest China. This nature reserve mainly focuses on the protection of extremely arid desert ecosystems and their associated biodiversity. The area has a typical semi-arid continental climate, with considerable seasonal and diurnal temperature variations. The average annual temperature is 7.8–10 °C, and the average annual precipitation is no more than 52.0 mm, which is far less than the average annual evaporation of 2754.9 mm [40]. The soils are dark chestnut and sandy chestnut [47]. The vegetation is sparse and has a simple plant community structure, which is predominated by typical super-xerophytes, including *Ephedra przewalskii*, *Nitraria sphaerocarpa*, *Reaumuria soongorica*, *Salsola passerine*, and *Sympegma regelii*; these species are abundant and exhibit a heterogeneous distribution.

Plant materials were carried out in July 2019 and consisted of collecting leaf, stem, and root samples from 3 sampling plots, which contained 5 randomly distributed target plant species, namely, *E. przewalskii*, *R. soongorica*, *S. regelii*, *N. sphaerocarpa* and *S. passerine*. The distance between plots was at least 1 km. Within each sampling plot, 5 healthy plant individuals of each plant species were randomly selected, with each plant individual at least 100 m away from another individual. Stem samples were collected from the branches of each plant (0.1 to 0.6 cm in diameter) at the half-crown level [4]. In addition, all leaves from the sampled offshoot were collected to represent the leaf compartment (except in the case of *E. przewalskii* due to the fallen leaves). Before collecting the root samples, the upper layer of soil (approximately 1–5 mm) was removed to clear away litter, and the roots were subsequently obtained from a depth of 30 cm under the canopy of each plant. All samples were collected over a 1-day period to reduce any heterogeneity imparted by the climatic conditions and immediately placed in sterile Ziplock bags, labeled, and transported to the laboratory in an ice box. The five repeating subsamples collected from each plot were equally merged into one except the leaf samples of *E. przewalskii*, and a total of 42 plant tissue samples were analyzed in this study.

### 2.2. Molecular Analysis

The plant materials (leaves, stems and roots) were washed with deionized water and surface sterilized by consecutive immersion for 5 min in 70% ethanol, 2 min in 5% sodium hypochlorite, and 30 s in 70% ethanol, followed by three rinses in sterile distilled water. Subsequently, the treated samples were freeze dried using liquid nitrogen and stored at −80 °C for DNA extraction. The genomic DNA of the endophytic fungi from the plant samples was extracted using the Invisorb Spin Plant Mini Kit according to the manufacturer’s protocol (Stratec Biomedical AG, Birkenfeld, Germany). The final DNA concentration and purity were determined by a NanoDrop 2000 UV-vis spectrophotometer (Thermo Scientific, Wilmington, CA, USA), and DNA quality was checked with 1% agarose gel electrophoresis.

The fungal internal transcribed spacer 2 (ITS2) amplicon libraries were generated using a two-step PCR procedure [48]. First, amplification was carried out with the primers ITS1F [49] and ITS4 [50] to generate the entire ITS regions of fungi with a thermocycler PCR system (GeneAmp 9700, Applied Biosystems ABI, Foster City, CA, USA). The PCR reactions were performed in triplicate in 20 μL mixtures containing 4 μL of 5 ×FastPfu Buffer (TransGen, Beijing, China), 2 μL of 2.5 mM dNTPs (TransGen, Beijing, China), 0.8 μL of each primer (5 μM), 0.4 μL of FastPfu Polymerase (TransGen, Beijing, China), and 10 ng of template DNA. The PCRs were conducted using the following programme: 3 min of denaturation at 95 °C; 27 cycles of 30 s at 95 °C, 30 s for annealing at 55 °C, and 45 s for elongation at 72 °C; and a final extension at 72 °C for 10 min. The product of the first PCR dilute solution was used as the template for the second PCR under the same conditions as in the first procedure, but the specific primer pair of fITS7 [51] and ITS4 was used for the amplification of the ITS2 regions of fungi. The resulting PCR products were extracted from a 2% agarose gel and further purified using the AxyPrep DNA Gel Extraction Kit (Axygen Biosciences, Union City, CA, USA) and quantified using a QuantiFluorTM -ST system (Promega, Madison, WI, USA) according to the manufacturer’s protocol. The purified amplicons were pooled in equimolar amounts and paired-end sequenced (2 × 300 base pairs (bp)) using Illumina MiSeq platform (PE300, Illumina, San Diego, CA, USA) with standard protocols by Majorbio Bio-Pharm Technology Co., Ltd. (Shanghai, China).

### 2.3. Bioinformatics Processing

Raw FASTQ files were quality filtered with Trimmomatic and merged with FLASH to obtain valid and high-quality sequences on the basis of the following criteria: (i) The reads were truncated at any site receiving an average quality score <20 over a 50 bp sliding window. (ii) All reads were assembled according to their overlapping sequences longer than 10 bp, and sequences that could not be assembled were discarded. The maximum mismatch ratio of overlapping regions was 0.2. Samples were distinguished according to the barcodes. Operational taxonomic units (OTUs) were clustered according to a 97% similarity cut-off using UPARSE (version 7.0.1090, http://www.drive5.com/uparse/ accessed on 22 November 2019) with a novel ‘greedy’ algorithm that performs chimera filtering and OTU clustering simultaneously after dereplication and discarding all singletons [52]. The taxonomy of each representative sequence was analyzed with the RDP Classifier algorithm (http://rdp.cme.msu.edu/ accessed on 22 November 2019) against the UNITE (version 8.0, https://unite.ut.ee/ accessed on 22 November 2019) database using a confidence threshold of 70% [53,54]. To eliminate the effects of the different numbers of sequences among the samples on the identified fungal community, the number of sequences per sample was normalized to the smallest sample size using the subsample command in MOTHUR. Subsequently, rarefaction curves were assembled, and the alpha diversity indices of OTU richness, Shannon diversity and evenness were calculated. The relative abundance of specific fungal taxa on the basis of OTU, order and class was defined as the number of reads of a particular taxon as a percentage of the number of all reads in a sample.

### 2.4. Statistical Analysis

All statistical analyses were implemented in R version 3.5.1. The Shapiro–Wilk test and Bartlett’s test were employed to check the normality and homoscedasticity of the data, respectively [17]. As the data of endophytic fungal OTU richness did not satisfy the normality of distribution and homogeneity of variance, the effects of plant species and tissue niche on the alpha diversity estimates for the fungal assemblage were examined using the Kruskal–Wallis (KW) test [48], followed by Welch’s tests for paired comparisons between samples [55]. Nonmetric multidimensional scaling (NMDS) was used to visualize the community composition dissimilarities of the endophytic fungi based on the Bray–Curtis, and the analysis of similarity (ANOSIM) was used to examine significant differences based on 999 permutations [56]. The permutational multivariate analysis of variance (PERMANOVA) was applied to test the variations in endophytic fungal community as explained by plant species and compartment niches [57]. The numbers of OTUs that were shared between plants and tissues were visualized using Venn diagrams. The relative abundances of abundant endophytic fungal OTUs in the different plant species and tissues were depicted using the pheatmap function; then, differential abundance analysis was performed with the KW H test (*p* < 0.05), and STAMP software (v.2.1.3) was used [58].

According to Toju et al. [59], we evaluated the host/tissue–fungus preference based on the interaction specialization index (*d′*) using the ‘dfun’ function in the R bipartite package. The *d’* index measures how strongly a fungus deviates from a random choice among plant partners that are available at a study site [60]. Considering the difficulty of estimating the host preferences of rare fungi, the *d′* estimates of the abundant endophytic fungal OTUs (>1000 reads) were used. Firstly, the sample data matrix were binarized to a sample-level matrix (present–absent data) and then converted into a species-level matrix, in which rows denote plant species, columns represent fungal OTUs, and cell entries indicate the number of samples from which respective combinations of plants and fungi were observed. Subsequently, the randomized species-level matrices were obtained based on 1000 permutations by shuffling the plant species labels in the sample fungal OTU matrix. The *d′* value of each fungal OTU was standardized as follows [61]: standardized *d′* = [*d′*_observed_ − Mean(*d′*_randomized_)]/SD(*d′*_randomized_), where *d′*_observed_ is the *d′* estimate of the original data, and Mean (*d′*_randomized_) and SD (*d′*_randomized_) are the mean and standard deviation of the *d′* values of the randomized data matrices. The standardized *d’* value was also calculated for each plant species based on the original and randomized data matrices mentioned above. Additionally, the two-dimensional preferences (*2DP*) seen in a pair of a plant species (*i*) and a fungal OTU (*j*) were quantified based on the species-level original and randomized matrices to evaluate to what extent each pair of each plant–fungus association was observed (counts) more or less frequently than would be expected by chance; *2DP* (*i, j*) = [*N*_observed_ (*i, j*) – Mean (*N*_randomized_ (*i, j*))]/SD (*N*_randomized_ (*i, j*)), where *N*_observed_ (*i, j*) is the number of the samples from which a pair of a plant–fungal OTU association was observed in the original data, and Mean (*N*_randomized_ (*i, j*)) and SD (*N*_randomized_ (*i, j*)) are the mean and the standard deviation of the number of samples for the focal plant–fungal OTU pair across randomized matrices. The *p* value obtained from the preference analysis was adjusted based on the false discovery rate (FDR) [62]. Similarly, tissue × fungal OTU matrix was carried out, and the standardized *d’* value of both each niche tissues and each fungal species was also calculated, as well as the *2DP* estimators.

Network analysis was performed to identify the microbial co-occurrence patterns across the three plant tissues associated with the five plants, respectively. Before network construction, we removed very rare OTUs and the abundant endophytic fungal OTUs (>1000 reads) were used [17,63]. Spearman’s rank correlations were calculated with all possible OTU pairs and robust correlations with correlation coefficient above 0.5 and statistical significance (*p* < 0.01) were noted [64]. The nodes of the network represent individual OTUs that have at least one significant co-occurrence relationship with other OTUs. The networks were explored and visualized with Cytoscape v3.5.1, and NetworkAnalyzer tool was used to calculate network topology parameters [65]. Structural attributes of networks such as the number of nodes and edges, graph density, network connectivity, the clustering coefficient, network centralization, characteristic path length and network heterogeneity were determined. Modular structure and groups of highly interconnected nodes were analyzed using the MCODE application with standard parameters [65,66]. A module is a group of nodes connected more densely to each other than to other nodes outside the group. Possible keystone species were those that demonstrated the higher degree values [67].

## 3. Results

### 3.1. Characterization of Illumina Sequencing Data

**Figure 1:**
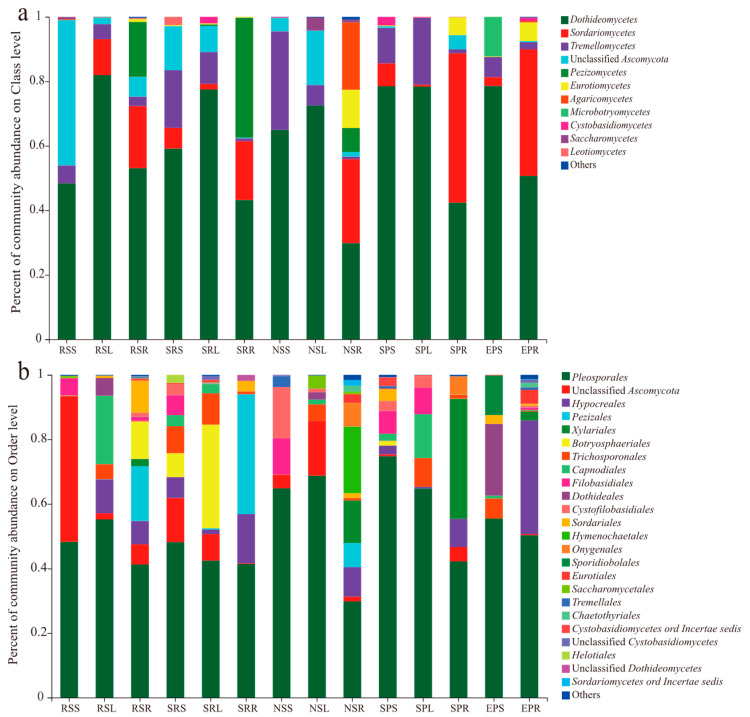
Relative abundance of endophytic fungi on class (**a**) and order (**b**) level in desert plants. The fungal class and order represents <0.01% of the total reads of endophytic fungi were all assigned to “Others”. RS, *Reaumuria soongorica*; SR, *Sympegma regelii*; NS, *Nitraria sphaerocarpa*; SP, *Salsola passerina*; EP, *Ephedra przewalskii*. S, stem; L, leaf; R, root.

After removing sequences of low quality, potential chimeras and singletons, the remaining non-chimeric fungal internal transcribed spacer 2 (ITS2) sequences (2,783,159 in total) were clustered into 504 operational taxonomic units (OTUs) at a 97% sequence similarity level. Of these 504 OTUs, 421 (2,692,818 reads) were identified as fungal OTUs. We then excluded the OTUs with <10 reads from all the samples, and the number of sequences per sample was normalized to the smallest sample size (39,948 reads), ultimately resulting in a normalized dataset composed of 337 fungal OTUs (1,677,816 reads). The identified fungal OTUs included 280 *Ascomycota*, 54 *Basidiomycota*, and 3 *Rozellomycota*, representing 3 phyla, 20 classes, 49 orders, 102 families, 163 genera and 219 species. A total of 124 relatively abundant OTUs (>1000 reads) accounted for 99% of the reads for endophytic fungi (Table S1). The DNA sequences of *Dothideomycetes* (29.84–81.92%) were the most abundant at the class level, and those of *Pleosporales* (29.83–74.76%) were the most abundant at the order level (Figure 1a,b). The members of *Sordariomycetes* (18.27–46.34%) were mainly distributed in root niches, while members of *Tremellomycetes* (2.21–30.59%) and unclassified *Ascomycota* (0.56–45.07%) were mainly distributed in the leaf and stem (Figure 1a). Rarefaction curves for the Sobs index at the OTU level across all samples approached an asymptote and showed that the overall fungal diversity was well represented (Figure S1).

### 3.2. Alpha and Beta Diversities

**Figure 2:**
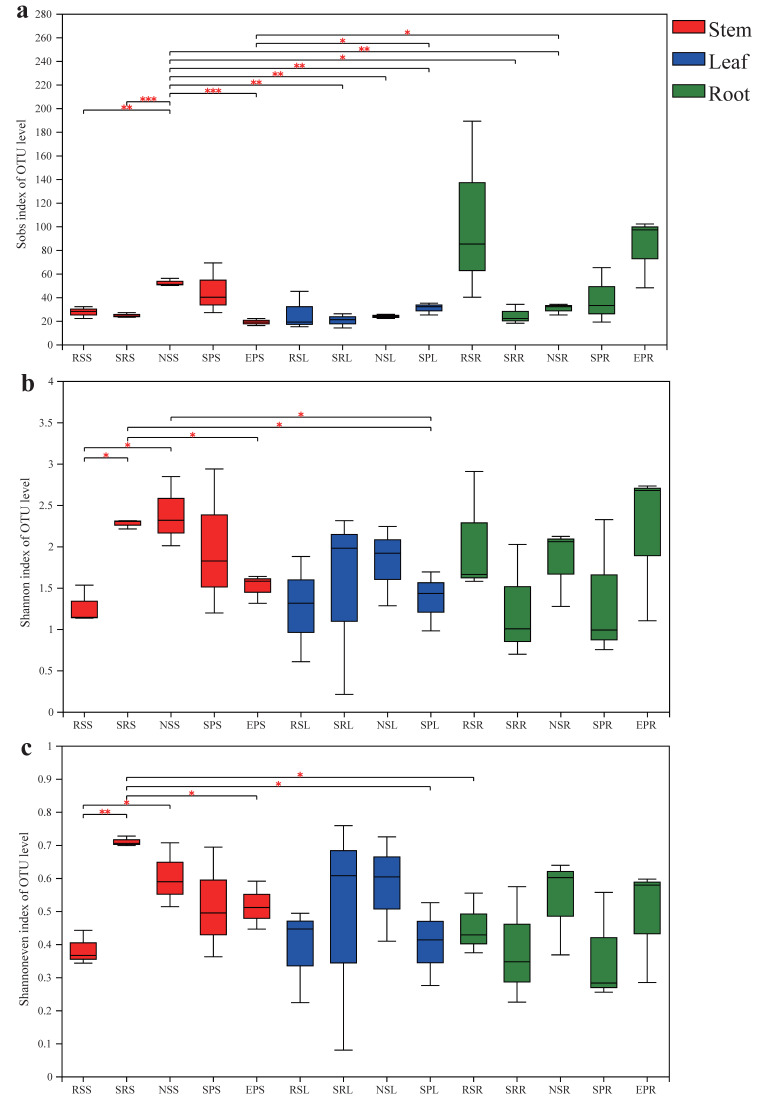
Operational taxonomic unit (OTUs) distribution across host plants and tissue niches. (**a**) OTU richness estimated by sobs index; (**b**) Shannon diversity; (**c**) Shannon’s evenness. Venn diagram displaying the overlap in OTUs composition between the different hosts and plant niches. RS, *Reaumuria soongorica*; SR, *Sympegma regelii*; NS, *Nitraria sphaerocarpa*; SP, *Salsola passerina*; EP, *Ephedra przewalskii*. S, stem; L, leaf; R, root. Asterisks indicates significance levels, * *p* < 0.05, ** *p* < 0.01 and *** *p* < 0.001.

Sobs index values were calculated to describe the observed OTU richness. Alpha diversity, including the Shannon diversity index and evenness, was analyzed based on OTU richness (Figure 2). The OTU richness of endophytic fungi in the stems, leaves and roots ranged between 19.0–52.3, 20.3–30.7 and 24.7–104.7 (mean values), respectively, and the root fungal richness in *R. soongorica* (104.7) and *E. przewalskii* (82.3) were much more diverse than other samples (Figure 2a). The Kruskal–Wallis test revealed that plant species had a significant effect on the OTU richness of endophytic fungi in the stem (χ^2^ = 10.939, *p* = 0.027), but not on that in the leaves (χ^2^ = 3.308, *p* = 0.347) and roots (χ^2^ = 5.957, *p* = 0.114). In addition, the integrated OTU richness was highly dependent on plant compartment effects (χ^2^ = 6.612, *p* = 0.037). In terms of the Shannon diversity and evenness estimates, we also found significant variation in the stem organs among the five plant species (*p* < 0.05) (Figure 2b,c).

**Figure 3:**
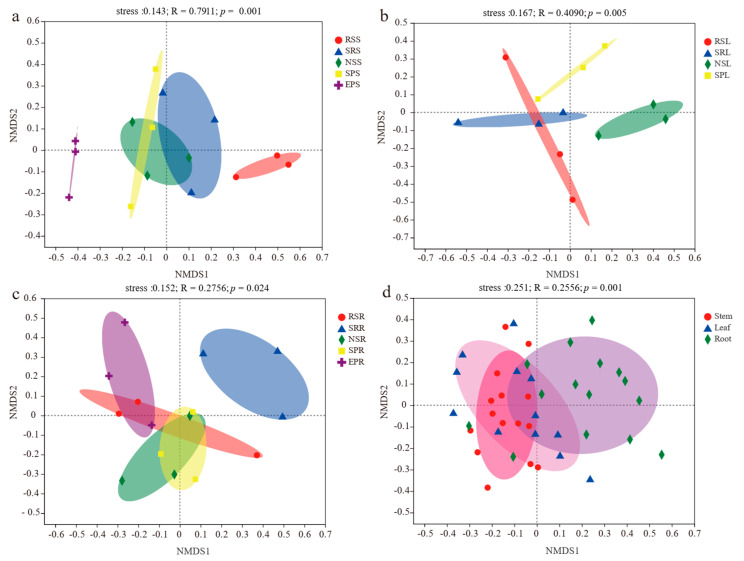
Nonmetric multidimensional scaling (NMDS) ordination of the fungal community composition: (**a**) Stem endophytic fungi; (**b**) Leaf endophytic fungi; (**c**) Root endophytic fungi; (**d**) Endophytic fungi in different tissue niches. The dissimilarities of endophytic fungi were based on the Bray–Curtis method and the nonparametric ANOSIM test (an analogue of univariate ANOVA) was used to examine the significant difference based on 999 permutations. Ellipses in the plots represent the grouping interval of endophytic fungi in different plant species (**a**–**c**) and tissue niches (**d**). RS, *Reaumuria soongorica*; SR, *Sympegma regelii*; NS, *Nitraria sphaerocarpa*; SP, *Salsola passerina*; EP, *Ephedra przewalskii*. S, stem; L, leaf; R, root.

Nonmetric multidimensional scaling (NMDS) revealed a significant effect of plant species and tissue niches on fungal community composition (Figure 3). The endophytic fungal community composition in stem, leaf and root significantly differed among the five plant species (stem: F = 0.7911, *p* = 0.001; leaf: F = 0.4090, *p* = 0.005; root: F = 0.2756, *p* = 0.024), and presented characteristics of permutation dispersion with higher heterogeneity of the OTU distribution in different hosts (Figure 3a–c). Plant species explained 51.4% of variability in stem fungal community composition (PERMANOVA, *p* = 0.001), which is higher than that in leaf (41.1%; PERMANOVA, *p* = 0.004) and root (38.7%; PERMANOVA, *p* = 0.014) fungal communities (Table 1). Moreover, the Bray–Curtis method revealed a significant effect of the plant compartment (F = 0.2556; *p* = 0.001), and the stem and leaf niches were clearly distinguished from the root tissues but did not cluster completely according to their respective niches (Figure 3d). The variation in fungal community explained by compartment niche accounted for 10.6% (PERMANOVA, *p* = 0.001) (Table 1).

### 3.3. Community Composition of Endophytic Fungi Across Hosts and Organ Compartments

The endophytic fungal communities in different organ niches have strong responses to plant species (Figure S2). *R. soongorica* represented the highest percentages of unique OTUs in the leaf (29.08%) and root (36.30%), and only seven OTUs (1.16 and 3.57%) were shared by the five plant species (Figure S2b,c). In addition, *N. sphaerocarpa* and *S. passerina* exhibited the highest proportion of unique OTUs (26.97 and 28.29%) in the stem samples, with 16 shared OTUs (5.26%) occurring among plant species (Figure S2a). Furthermore, 81 OTUs (12.05%) were shared by the leaf, stem and root niches, with the root exhibiting the highest percentage of unique OTUs (41.54%) (Figure S2d). Notably, the OTUs of leaves (10.36%) and stems (10.48%) shared greater proportion of taxa with roots than with each other (2.46%).

**Figure 4:**
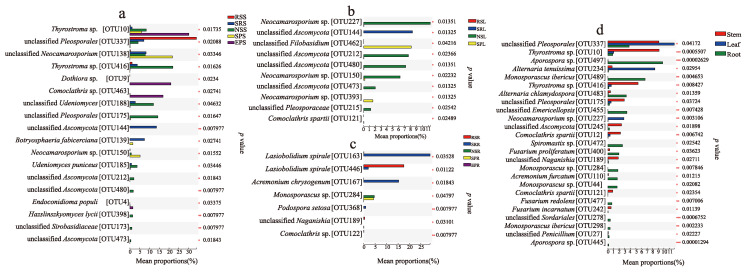
Differential abundance analysis of abundant fungal OTUs. (**a**) Stem endophytic fungi; (**b**) Leaf endophytic fungi; (**c**) Root endophytic fungi; (**d**) Endophytic fungi in different tissue niches. Kruskal–Wallis H test were performed to compare the significant differences of OTUs abundance among plant species and tissues. Asterisks indicates significance levels, * *p* < 0.05, ** *p* < 0.01 and *** *p* < 0.001. Only differentially abundant OTUs (*p* < 0.05) are shown. RS, *Reaumuria soongorica*; SR, *Sympegma regelii*; NS, *Nitraria sphaerocarpa*; SP, *Salsola passerina*; EP, *Ephedra przewalskii*. S, stem; L, leaf; R, root.

Clustering heatmap based on the top 50 most abundant OTUs revealed varied occurrence patterns of fungal OTUs among the different plant species (Figure S3a–c). The relative abundances of abundant OTUs in the stem, leaf and root significantly differed among plant species (*p* < 0.05), with the most fungal richness differences occurring in the stem niche (Figure 4a). The taxa of OTU337 (unclassified *Pleosporales*) was the most abundant fungus in the stem of *R. soongorica*, OTU138 (*Neocamarosporium*) was most abundant in *S. passerina*, while OTU144 (unclassified *Ascomycota*) and OTU139 (*Botryosphaeria*) were more abundant in *S. regelii* (Figure 4a); for *N. sphaerocarpa*, OTU416 (*Thyrostroma*), OTU175 (unclassified *Pleosporales*) and OTU188 (*Udeniomyces*) were significantly abundant, and OTU10 (*Thyrostroma*), OTU9 (*Dothiora*) and OTU463 (*Comoclathris*) were most abundant in *E. przewalskii* (Figure 4a). For leaf and root niches, the differences in taxonomic identity and relative abundance of the fungal taxa for each plant species were less than those of the stems (Figure 4b,c). The significant differences observed for fungal abundance in the leaf compartment was explained by higher relative abundances of OTU462 (*Filobasidium*) in *S. passerina*, OTU144 (unclassified *Ascomycota*) in *S. regelii* and *Neocamarosporium* (OTU227 and OTU150) and unclassified *Ascomycota* (OTU212 and OTU480) in *N. sphaerocarpa* (Figure 3b). For root, OTU446 (*Lasiobolidium*) was the most abundant in *R. soongorica* together with OTU163 (*Lasiobolidium*) and OTU167 (*Acremonium*) for *S. regelii*; OTU284 (*Monosporascus*) was shared and significantly abundant in *N. sphaerocarpa* and *S. passerina* (Figure 4c).

The heatmap also displayed the biased occurrence of endophytic fungi in above- (stem and leaf) and below- (root) ground compartments, and the accompanying clustered stem and leaf OTUs significantly differed from those of the root’s habitats (Figure S3d). The more in-depth analysis revealed root niches occupied the most abundant fungal OTUs differed from stem and leaf niches (Figure 4d). For example, *Aporospora* OTU497, *Alternaria* OTU483, *Emericellopsis* OTU455, *Spiromastix* OTU 472, *Fusarium* OTU400, *Acremonium* OTU110, and *Monosporascus* (OTU489, OTU284 and OTU44) were the most abundant in root; while OTU337 (unclassified *Pleosporales*), OTU234 (*Alternaria*) and OTU227 (*Neocamarosporium*) were most abundant in leaf, and OTU10 and OTU416 (*Thyrostroma*), OTU175 (unclassified *Pleosporales*), OTU245 (unclassified *Ascomycota*), OTU12 (*Comoclathris*) and OTU189 (*Naganishia*) were abundant in stem (*p* < 0.05) (Figure 4d).

### 3.4. Host and Organ Preferences of Endophytic Fungi

**Figure 5:**
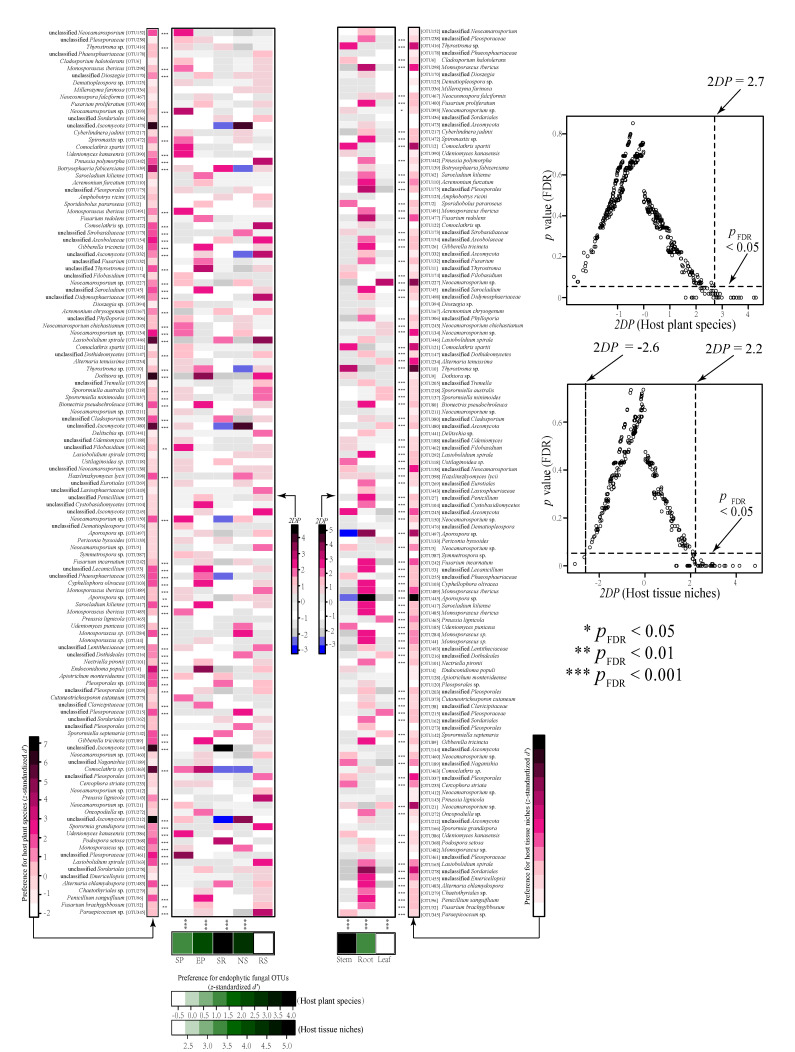
Analysis of host-tissue/fungus preferences. The standardized *d’* estimate of preferences for fungal operational taxonomic units (OTUs) are shown for each of the hosts and tissue niches (column). Likewise, the standardized *d’* estimate of preferences for plant species and tissue niches are indicated for each of the observed fungal OTUs (row). Each cell in the matrix indicates a two-dimensional preference (*2DP*) estimate, which evaluates to what extent each pair of each plant/tissue–fungus association was observed (counts) more or less frequently than would be expected by chance. The *p* values were adjusted based on the false discovery rate (FDR). RS, *Reaumuria soongorica*; SR, *Sympegma regelii*; NS, *Nitraria sphaerocarpa*; SP, *Salsola passerina*; EP, *Ephedra przewalskii*.

Host–organ/fungus preference analysis showed that four plant species (*S. regelii*, *N. sphaerocarpa*, *S. passerina*, and *E. przewalskii*) had significant preferences for endophytic fungi, but all organ niches (stem, root and leaf) presented significant preferences for endophytic fungi (Figure 5). Alternatively, 49 out of 124 endophytic fungal OTUs displayed both host and organ preferences. In addition, 21 endophytic fungal OTUs were exclusively displayed significant host preferences, such as OTU139 (*Botryosphaeria fabicerciana*), OTU473, OTU144 and OTU212 (*Ascomycota*), OTU446 (*Lasiobolidium spirale*), OTU9 (*Dothiora*) and OTU463 (*Comoclathris*), whereas 39 endophytic fungal OTUs were exclusively displayed significant organ niches preferences, such as OTU12 (*Comoclathris spartii*), OTU110 (*Acremonium furcatum*), OTU477 (*Fusarium redolens*), OTU138 and OTU21 (*Neocamarosporium*), OTU245 (*Ascomycota*), OTU497 (*Aporospora*) and OTU278 (*Sordariales*) (Figure 5). In our dataset, 26 pairs of plant species and endophytic fungi showed significantly strong preferences (two-dimensional preferences (*2DP*) > 2.7), such as the pairs of *E. przewalskii* with OTU9 and OTU4 (*Endoconidioma populi*), *S. regelii* with OTU144 (*Ascomycota*), *S. passerina* with OTU461 (*Pleosporaceae*), and *N. sphaerocarpa* with OTU212, OTU480 and OTU473 (*Ascomycota*) (Figure 5). In contrast, 44 of 372 organ and endophytic fungus pairs were observed to show significantly strong preferences (*2DP* > 2.2). Among them, 33 pairs revealed strong preferences between the root niche and endophytic fungi, such as OTU298 (*Monosporascus ibericus*), OTU175 (*Pleosporales*), OTU417 (*Sarocladium kiliense*), OTU477 (*Fusarium redolens*) and OTU445 (*Aporospora*) (Figure 5).

### 3.5. Co-Occurrence Networks of Endophytic Fungi

**Figure 6:**
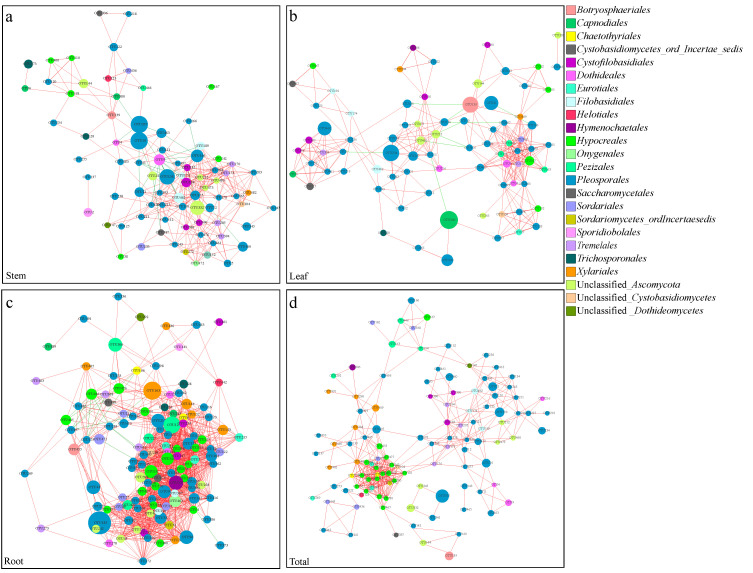
Co-occurrence networks of microbial taxa in the fungal communities. (**a**) Stem endophytic fungi; (**b**) Leaf endophytic fungi; (**c**) Root endophytic fungi; (**d**) Total endophytic fungi. Nodes represent fungal OTUs, whereas edges represent significant interactive correlations between pairs of OTUs. Node color represents the order of fungal OTUs and the size of nodes corresponds to the relative abundances of specific fungus. Red edges indicate positive relationships, and green edges indicate negative relationships. The thickness of each edge is proportional to the *p* values.

The root co-occurrence network showed the highest network connectivity, exemplified by the highest numbers of nodes (120) and edges (1385) (Figure 6a–c and Table S2). The root network also had significantly higher mean values of network density (0.194 versus 0.098 and 0.100) and centralization (0.247 versus 0.181 and 0.134) than the stem and leaf networks, while the clustering coefficient for the leaf habitat was higher than those for the stem and root networks (Table S2). MCODE analysis returned several significant clusters of fungal groups (six in stems, six in leaves and seven in roots) that were particularly closely connected in the network (Figure S4a–c), indicating the typical small-world characteristic of the three networks in this arid desert. Notably, the analysis of the relationships between fungal OTUs in the entire dataset revealed a high percentage of co-presence rather than mutual exclusion (Figure 6a–c).

To take into account the interactions between the above- and belowground fungal communities, an integrated co-occurrence network was constructed to interpret the relationships among the fungal communities across the different organs (Figure 6d). The construction of this network led to the identification of 112 nodes and 441 edges, with the highest values of characteristic path length and network heterogeneity (Figure 6d; Table S2). The fungal nodes in the network tended to be shared by all three niche habitats (51.79%), which confirmed the results in terms of the relationships and overlap among the different plant tissues. The nodes shared by leaves–roots and stems–roots accounted for 14.29 and 11.61% of the total, respectively, whereas 21.43% of the nodes appeared to be specific to the root niche habitat.

According to the number of connections established with the rest of the network, 10 dominant keystone OTUs were arbitrarily identified here based on the number of degrees for each node (Table S3). Although OTUs from *Pleosporales* were the predominate constituent nodes in all the networks, which accounted for 46.91% of the total nodes in stems, 51.90% in leaves, 39.17% in roots and 41.96% in the integral network, the taxa with a high number of degrees but relatively low abundance served as important nodes in terms of maintaining the function and structure of the microbial community (Table S3). There were no overlapping keystone OTUs found in the networks among the different plant compartments (except OTU9 in leaves and roots). In order level, the keystone species of *Cystofilobasidiales* and three unclassified *Ascomycota* were exclusively found in the stem network, and *Sordariales* and *Pezizales* only appeared in the leaf network (Table S3). Members of *Hypocreales* played important central roles in connecting the above- and belowground fungal networks, and MCODE analysis also returned the *Hypocreales* groups that produced the rank 1 cluster with 20 nodes and 171 edges (Figure S4d and Table S3).

## 4. Discussion

### 4.1. Fungal Community Associated with Xerophytic Desert Shrubs

Desert shrubs play a crucial role in sustaining and restoring fragile desert ecosystems. Previous studies have focused on plant communities, ecological adaptability and mechanisms for drought adaptation of these plants, such as *R. soongorica*, *S. regelii* and *N. Sphaerocarpa* [2,3,68]. However, desert plants interact with diverse clades of endophytic fungi in their internal organizations to maintain their adaptation and ecological success in arid habitats [69]. Although a large number of endophytic fungi have been isolated from plants in arid environments [1,7,9,70,71,72], few studies have focused on the molecular phylogenetics of the fungal microbiome in desert plants, especially the extremely xeric shrubs. To the best of our knowledge, this study is the first to report the fungal community assembly associated with typical super-xerophytic plant communities in extremely arid desert. Here, a total of 337 OTUs of endophytic fungi were obtained at a 97% sequence similarity level. This level of endophytic fungal diversity in our study associated with the xeric shrubs in Anxi extremely arid desert, Northwest China was relatively lower than that associated with plants in subtropical forests, farmland plantations and even wetland ecosystems [30,38,48]. Considering the unique habitat provided by arid areas, it seems that the typical harsh environmental conditions of extreme desert lands truly reduce the number of plant endophytes. However, compared to other studies in similar extreme environments [26,73,74], the diversity of these microbial communities in our study is still relatively high in this arid desert, which may be associated with microenvironmental heterogeneity that is characteristic of the arid environment. Massimo et al. [9] also demonstrated that fungal endophytes in desert plants were highly diverse and distinctive symbionts but infrequent in culture. Therefore, the methodological processing may also result in the loss of some taxa that were initially below detection levels. Additionally, the unidentified taxa presented in our study also highlight the insufficient research on desert plants and fungal resources, and further exploration is needed. These suggest that the endophyte diversity in the desert shrubs of the extremely arid desert may still be underestimated.

Our study showed that *Ascomycota* was dominant among the desert plants. Most members of *Ascomycota* are saprophytic and, therefore, the main decomposers in habitats. The predominance of *Ascomycota* seems to be characteristic of the endophytic mycota identified from other plant species [72,75,76]. However, Wenndt et al. [77] investigated the endophytic fungal communities from perennial bunchgrass *Stipagrostis sabulicola* in the Namib Sand Sea, and demonstrated that the dryland grass tended to harbor endophytic communities composed predominantly of potential saprophytes, which can decompose large amounts of plant litter immediately after senescence. Therefore, we hypothesize that the permission presence of desert plants for more saprophytic fungi may maximize the possibility of in situ recovery of plant bound nutrients and promote the nutrient recycling in desert soil [77,78,79]. In the present study, *Pleosporales* (29.83–74.76%) were most abundant at order level, which was consistent with previous studies of endophytes in the arid or desert habitats [18,26,77,80,81]. The taxon of *Pleosporales*, the largest order of *Dothideomycetes* [82], is one of the most represented orders in arid and semiarid areas [83]. Most of the endophytic fungi, especially root-associated fungi, isolated from previous studies on desert plants, belong to the order of *Pleosporales* [1,42,44,45]. The keystone species represented in the root fungal network in our study were also identified as *Pleosporales.* The wide distribution of *Pleosporales* members among desert plants and the identification of keystone species suggest that this particular fungal group occupies a crucial role in maintaining the structure and stability of the desert fungal network.

The five target plants in our study belong to families of *Chenopodiaceae* (*S. passerina* and *N. sphaerocarpa*), *Tamaricaceae* (*R. soongorica*), *Zygophyllaceae* (*S. regelii*) and *Ephedraceae* (*E. przewalskii*), with these plant communities are found in desert, beach, arid grasslands and other different environments. Compared with these phylogenetically related plants, the orders of *Hypocreals*, *Xylariles*, *Onygenales*, *Eurotiales* and *Saccharomycetales* have been reported in coastal *Chenopodiaceae* plants [84,85]; *Botryosphaeriales*, *Capnodiales*, and *Dothideales* were found in the steppe ecosystem of *Chenopodiaceae* and *Tamaricaceae* plants [86]; and *Pezizakes*, *Xylariles*, *Botryosphaeriales*, *Sordariales* and *Hymenochaetales* have been identified in *Chenopodiaceae*, *Zygophyllaceae* and *Ephedraceae* plants in forest ecosystems [87,88,89]. This suggests the plant species associated with the same phylogenetic levels may play a selective recruitment role in endophytic communities and tend to share the same microflora regardless of their distributive distance [20]. Nevertheless, compared with previous studies in similar desert environment, the orders of *Eurotiales*, *Chaetothyriales*, *Capnodiales*, *Dothideales*, *Hymenochaetales*, *Hypocreales*, *Xylariales* and *Sporidiobolal* have been reported to be common species in arid or semiarid desert plants, such as *Aloe vera*, *Welwitschia mirabilis*, *Tecomella undulate*, *Dactyloctenium scindicum* and even *Nicotiana* and *Cactaceae* plants [8,18,20,26,80,90]. Additionally, most endophytes isolated from the desert plants in Northwest China belongs to *Pleosporales* and *Hypocreales* [26,27]. Environments, considered as the primary origin of the plant microbiome, contain most microorganisms required for the assembly of the plant microbiome [91]. Thus, the relatively consistent environmental conditions in desert habitats may explain the certain degree of similarity in plant microflora. In this study, however, *Cystofilobasidiales* and *Trichosporonales* were reported in Anxi extremely arid desert for the first time. The taxa of *Dioszegia*, *Botryosphaeria*, *Filobasidium*, *Cyphellophora*, *Endoconidioma*, *Apiotrichum*, *Udeniomyces*, *Podospora* and *Lasiobolidium* were highly exclusively specific to the selected xeric shrubs, which indicated the particular location and the species collected significantly shaped the uniqueness of theses fungal groups. This may be related to the processes associated with the morphological, physiological and genetic traits of the plant species. Furthermore, the differentiation of members of *Sordariomycetes* and *Tremellomycetes* in above- and belowground tissues could possibly be the result of utilization of resources by fungal microbes, which drives the rapid evolutionary radiation of microorganisms competing for resources, causing them to diverge and adapt to different niches to reduce competition in arid environments [13,92].

### 4.2. Host Selection Shape Fungal Assembly in Each Compartment Niches

Despite the fact that shrubs in the same arid habitat seem to receive similar fungal propagules [15,93], our study revealed shrub species strongly shaped the community composition of endophytic fungi in each compartment niches, as suggested by the results from the heatmap and ANOSIM analysis of fungal community compositions. We found that plant species significantly affected the OTU richness of the stem endophytic fungi, and the endophytic fungi in stem showed higher variability among the five plant species than leaf and root, which was consistent with Fonseca-García et al. [20], who investigated the microbiome of *Cactaceae* plants and found that the plant host plays a larger role in fungal communities of the stem endosphere. We suspected that this may be related to the ability of endophytic fungi to penetrate the plant endodermis, the pericycle and even the xylem vessels [4,13,94]. PERMANOVA analysis also showed more explanation of host effect on stem fungal composition than leaf and root. This is probably reasonable as the surfaces of leaves or roots were supposed to be the interface between the host and the environment, thus the microbes inhabiting the two niches were also influenced by environment factors, especially the root-associated fungi. Nevertheless, our findings evidenced the strong selection pressures caused by host immune system to allow relatively fungal endophytes to penetrate and colonize [13]. Differences in host chemistry and nutrient contents could also fundamentally influence endophytic fungal colonization and consequently promote differences in the dominant members of the fungal community [48,95,96].

The host/fungus preference analysis further supported the host selection effect mentioned above and demonstrated the two-dimensional host–fungus preference. To a certain extent, the enrichment and depletion of specific endophytic microbiomes are not passive processes but rather depend on the active selection of microbial consortia by the plant host [4,97,98]. The ability of endophytic fungi to successfully colonize a host plant requires the presence of specific traits, such as the production of cell-wall-degrading enzymes, and intricate interplay between the fungi and the host plant’s innate immune system [4,94,99]. However, of the five plants, *R. soongorica* showed no significant preferences for endophytic fungi. Kemler et al. [90] investigated the foliar fungi of the desert plant *Welwitschia mirabilis* and indicated the leaf-associated fungi showed no specialisation within their unique host. This research suggested certain fungal communities may not co-evolve with the host but primarily established by inoculation over long distances in space in the desert habitats with little plant coverage. Based on the information, the transmission mechanism by which specific associations form between host plants and the endophytes remains to be determined.

### 4.3. Organ Differentiation and Tissue-Specificity of Endophytic Fungi

Previous studies have documented differences in the above- and belowground tissues of endophytic fungi in desert environments [18,20,26,27]. In our study, the heatmap and NMDS analysis revealed clustering leaf and stem fungal communities, which differentiated significantly from root tissue niches. This suggests the obviously above- and belowground niche differentiation and the essential important of root niches on desert plants fungal microbiome. The light intensity, humidity and temperature of the desert habitats in which the different microbes survive and the chemical activity of the above- and belowground tissues against fungal infections can absolutely lead to differentiation in the endophytic fungal communities [100,101]. Moreover, the deeply and extensively distributed underground root systems of desert plants provide more hosts and substrate for infection by endophytic fungi than aboveground tissues [99]. Intriguingly, we found that the leaves and stems showed a greater percentage of shared taxa with those in the root habitat than with each other. Considering that the endosphere niches might select microbial taxa from the nearby species pool, the taxonomic overlap between root and aboveground compartments in our result indicates that the root endophytes can be transported to plant above-ground compartments via internal plant organ transmission [13]. Zarraonaindia et al. [102] investigated the *Vitis vinifera*-associated microbiota and found that the microbiota of the leaves, flowers, and grapes shared greater proportions of taxa with the belowground communities than with each other, suggesting that soil served as a common reservoir for plant microbiota. Soil harbors an extraordinarily rich diversity of microorganisms and largely determines the initial inoculum of endophytic root-associated microbiota [93,97,103]. In the studied arid desert, the abundant root endophytes may also provide an inoculum source, and then gradually enriched and filtered at stem and leaf compartment niches.

Each of the plant ecological niches provides relevant biotic and abiotic gradients, such as in the availability of soluble organic compounds [4,48,97], and the different ecological processes can also contribute to the high degree of compartment specificity [25,104]. In our study, all tissue niches presented significant preferences for endophytic fungi and PERMANOVA analysis also revealed significant effect of compartment niches on fungal community composition. This is consistent with the previous research of Fonseca-García et al. [20], who showed that the microbiome assemblies of *Cacti* plants in arid ecosystems were primarily influenced by the plant compartment than by host plants. However, the study was limited to host plants at the same level of phylogeny. In our study, the influence of plant identity at different species levels was more significant than at the plant tissue compartment. Thus, we speculated that plant tissue niches may play a more predominant role in modifying the microbial community at the same phylogenetic plant species level. Individual tissue niches as distinct substrates could reflect preferences for dominant taxa [105]; simultaneously, different endophytic fungi also vary in their capacity for utilizing or surviving on a specific substrate [106,107]. For instance, the deposition of large amounts of carbon in root tissue, such as root exudates, could directly drive the colonization of root-associated fungi [48,108]. Some leaf endophytic fungi have been shown to harbor corresponding receptors to ensure survival in the phyllosphere environment [48,109].

### 4.4. Co-Occurrence Network and Keystone Species

Co-occurrence analysis revealed that the root niche showed the highest network connectivity and interactions, suggesting that the root endophytic fungal network is more stable and functional than the stem and leaf networks in this arid desert. He et al. [30] indicated that the interspecies relationships in fungal communities could be strengthened by upregulated drought or wetting disturbance. Thus, the strong fungal interactions in the root habitat are likely one of the factors explaining the reasonable adaptation of desert plants to this particular soil type. In the present study, MCODE analysis returned several significant clusters of fungal groups, indicating the typical small-world characteristic of the networks in the extremely arid desert. The links between OTUs in the different modules in the network can also contribute to more intensified interactions among fungal OTUs at a high organizational level of the network [30]. The edges in co-occurrence networks could represent positive or negative correlations and imply a biologically or biochemically meaningful relationship between fungal species [17,63]. The relationships between fungal OTUs in the entire dataset revealed a high percentage of copresence in our study. In arid deserts, fungal assemblages may persist in the community by establishing reciprocal symbioses with each other to resist stressful conditions [17,48,110]. The disconnected microhabitats and the presence of a large number of dormant cells in the bulk sand can also explain the identification of a dominance of co-presence interactions in the arid desert [8,111].

Keystone species are represented by the centralized nodes in the network, have a large influence on ‘information’ transfer throughout the community, and act to stabilize the microbial community [112,113]. We found that taxa represented by nodes with a high number of degrees but relatively low abundance were important in maintaining the function and structure of the microbial community (Table S3). This finding is in agreement with those from a study by Mandakovic et al. [110], who compared microbial co-occurrence networks representing soil bacterial communities along a western slope of the Andes in the Atacama Desert and revealed that highly abundant OTUs were not as important as some of the relatively rare but highly connected species in terms of network dynamics and stability. Xue et al. [33] demonstrated that the removal of keystone taxa from microbial co-occurrence networks significantly decreased the number of edges and average connectivity of the network. No overlapping keystone OTUs were found in the networks among the different plant compartments, indicating that role shifts of fungal OTUs occurred in the different plant tissue niches [30,35]. However, the keystone species originated from nearly the same taxa at a higher phylogenetic level; for example, members of the order *Pleosporales* were keystone species in root fungal networks, suggesting that the functional roles of nodes in the networks could be conserved at a higher phylogenetic level [30]. Most groups of *Pleosporales* isolated from arid areas also represented high potential sources for antifungal and antitumoral agents [80].

Among the identified keystone species in our study, the taxa of *Udeniomyces*, *Podospora*, *Lasiobolidium*, *Dioszegia*, *Dothiora*, *Thyrostroma* and *Sporormiella* were first reported in the desert ecosystem and exhibited specificity to our host plants. In addition, *Alternaria* was general isolated as the most dominant fungal genera in desert plants as well as other symptomless halophytes [9,26,114,115]. Although *Alternaria* is known as a phytopathogen of several plant species, this species was frequently reported as a dominant member of desert ecosystems and displayed a greater adaptive potential [71,76]. Furthermore, members of *Hypocreales*, *Chaetothyriales* (*Cyphellophora*) and *Eurotiales* (*Penicillium*) played important central roles in connecting the above- and belowground fungal networks. Abundant studies have focused on the entomopathogenic effect of *Hypocreales* strains and their potential use as biocontrol agents of insects [116,117,118]; thus, the important connections of this fungus in the co-occurrence network may be related to the biological defense mechanisms of desert plants. *Penicillium*, a well-known *Ascomycota* taxon that secretes antibiotics, is also known for its tolerance to low water potential and could develop strong relationships with other members in the module through antibiotics or secondary metabolites [119].

## 5. Conclusions

This study first revealed the variation in the plant organ niche levels of the fungal microbiome associated with xerophyte shrubs in an extremely arid desert ecosystem. Even in identical environmental conditions, we showed that host selection strongly drives the niche-based processes of endophytic fungal microbiome across the root, stem and leaf compartments. Fungal communities in root libraries were more diverse and may provide inoculum source, and then gradually enrich and filter at stem and leaf organ niches. The host’s preference and the organ specificity of desert endophytic fungi were demonstrated, and the unique specific taxa were also observed. The composition structure of fungal communities in root niches revealed more stable and functional than the stem and leaf as well as network complexity. Overall, this study provides a holistic understanding of ecological process of fungal endophytes associated with xerophyte shrubs across different plant organ niche levels. These efforts will lend a baseline to further deepen our knowledge of plant–microbe interactions in extremely arid desert environments.

## Figures and Tables

**Table 1:** Effects of plant species and compartment niches on fungal community structure based on PERMANOVA.

	Plant Species		Compartment Niches	
Niche	*R^2^* (%)	Pr (>*F*)	*R^2^* (%)	Pr (>*F*)
Stem	51.4	0.001	10.6	0.001
Leaf	41.1	0.004		
Root	38.7	0.014		

## Data Availability

The raw data sequences were deposited in the NCBI Sequence ReadArchive (SRA) under the Bioproject number PRJNA681429 and Bio Sample accession numbers SAMN16953820 to SAMN16953861.

## Supplementary Materials

The following are available online at https://www.mdpi.com/article/10.3390/jof7070578/s1, Figure S1: Rarefaction curves for the observed endophytic fungal operational taxonomic units (OTUs). Rarefaction curves were assembled showing the number of OTUs, defined at the 97% sequence similarity cut-off in MOTHUR, relative to the number of total sequences. RS, Reaumuria soongorica; SR, Sympegma regelii; NS, Nitraria sphaerocarpa; SP, Salsola passerina; EP, Ephedra przewalskii. S, stem; L, leaf; R, root, Figure S2: Operational taxonomic unit (OTUs) distribution across host plants and tissue niches. Venn diagram displaying the overlap in OTUs composition between the different hosts and plant niches. RS, Reaumuria soongorica; SR, Sympegma regelii; NS, Nitraria sphaerocarpa; SP, Salsola passerina; EP, Ephedra przewalskii. S, stem; L, leaf; R, root, Figure S3: Heatmap depicting the occurrences of relatively abundant fungal operational taxonomic units (OTUs, >1000 reads). a, Stem endophytic fungi; b, Leaf endophytic fungi; c, Root endophytic fungi; d, Endophytic fungi in different tissue niches. The color of each heat map cell indicates the relative abundance of the corresponding fungal OTUs. Cluster analysis was performed based on Bray–Curtis similarities. RS, Reaumuria soongorica; SR, Sympegma regelii; NS, Nitraria sphaerocarpa; SP, Salsola passerina; EP, Ephedra przewalskii. S, stem; L, leaf; R, root, Figure S4: Modular groups of highly interconnected nodes. MCODE analysis showing the sub-networks with highest node scores in different plant niches and the total networks, Table S1: Molecular identification of endophytic fungi (>1000 reads) at a 97% sequence identity level, Table S2: Structural attributes of networks obtained through network analysis for different plant niches (stem, leaf and root) and the total networks, Table S3: The ten keystone species in each plant niches, characterized by their number of total degrees, as either positive (+) or negative (−), and their relative abundance (%).
