# Supplementary material for: Fungal Endophytic Community and Diversity Associated with Desert Shrubs Driven by Plant Identity and Organ Differentiation in Extremely Arid Desert Ecosystem
Source: J Fungi (Basel). 2021 Jul 20;7(7):578. doi: 10.3390/jof7070578 (PMC8306007; doi:10.3390/jof7070578)
Supplement: Supplementary file 1 [file jof-07-00578-s001.zip › Supplementary materials/Table S2 and S3.pdf]

Table S2 Structural attributes of networks obtained through network analysis for different plant niches (stem, leaf and root) and the total networks.

	Stem	Leaf	Root	Total
Nodes	81	79	120	112
Edges	318	308	1385	441
Network diameter	8	7	7	11
Network connectivity	7.852	7.797	23.083	7.875
Clustering coefficient	0.450	0.627	0.595	0.525
Network density	0.098	0.100	0.194	0.071
Network centralization	0.181	0.134	0.247	0.139
Characteristic path length	3.101	3.453	2.357	3.913
Network heterogeneity	0.680	0.603	0.621	0.852
Co-presence (%)	91.5%	97.1%	98.9%	100%

Table S3 The ten keystone species in each plant niches, characterized by their number of total degrees, as either positive (+) or negative (-), and their relative abundance (%).

	Order	Genus	Species	OTU	degree	+	-	Abundance (%)
Stem	*Pleosporales*	*Neocamarosporium*	*Neocamarosporium* sp.	OTU150	22	21	1	1.1735
	Pleosporales	unclassified	unclassified *Pleosporales*	OTU175	19	19	0	2.9137
	Cystofilobasidiales	*Udeniomyces*	unclassified *Udeniomyces*	OTU188	19	17	2	2.9507
	Filobasidiales	*Filobasidium*	unclassified *Filobasidium*	OTU174	18	16	2	0.6769
	Cystofilobasidiales	*Udeniomyces*	*Udeniomyces puniceus*	OTU185	18	15	3	0.9139
	Pleosporales	*Hazslinszkyomyces*	*Hazslinszkyomyces lycii*	OTU398	17	17	0	0.2811
	Unclassified *Ascomycota*	unclassified	unclassified *Ascomycota*	OTU480	17	17	0	0.3411
	Unclassified *Ascomycota*	unclassified	unclassified *Ascomycota*	OTU212	17	17	0	0.3945
	Unclassified *Ascomycota*	unclassified	unclassified *Ascomycota*	OTU473	17	17	0	0.0959
	Tremellales	unclassified	unclassified *Sirobasidiaceae*	OTU173	16	16	0	0.1922
Leaf	*Pleosporales*	unclassified	unclassified *Phaeosphaeriaceae*	OTU178	18	18	0	0.0005
	Dothideales	*Dothiora*	*Dothiora* sp.	OTU9	18	18	0	1.6539
	Sordariales	*Podospora*	*Podospora setosa*	OTU368	18	18	0	0.0805
	Hypocreales	*Acremonium*	*Acremonium chrysogenum*	OTU167	18	18	0	3.2312
	Pezizales	*Lasiobolidium*	*Lasiobolidium spirale*	OTU446	18	18	0	0.0098
	Pezizales	unclassified	unclassified *Ascobolaceae*	OTU154	18	18	0	0.0031
	Tremellales	*Dioszegia*	unclassified *Dioszegia*	OTU170	18	18	0	0.0003
	Pleosporales	unclassified	unclassified *Didymosphaeriaceae*	OTU498	18	18	0	0.0015
	Pleosporales	*Alternaria*	*Alternaria chlamydospora*	OTU483	15	15	0	0.0736
	Pleosporales	unclassified	unclassified *Lentitheciaceae*	OTU493	15	15	0	0.1088
Root	*Filobasidiales*	*Naganishia*	unclassified *Naganishia*	OTU189	52	52	0	0.0921
	Pleosporales	*Thyrostroma*	*Thyrostroma* sp.	OTU10	51	51	0	0.8366
	Dothideales	*Dothiora*	*Dothiora* sp.	OTU9	49	49	0	0.0270
	Pleosporales	unclassified	unclassified *Pleosporales*	OTU337	47	45	2	3.7803
	Pleosporales	*Neocamarosporium*	*Neocamarosporium* sp.	OTU5	46	46	0	0.0092
	Pleosporales	*Thyrostroma*	unclassified *Thyrostroma*	OTU11	46	46	0	0.0182
	Pleosporales	unclassified	*Pleosporales* sp.	OTU120	45	45	0	0.0003
	Pleosporales	*Neocamarosporium*	*Neocamarosporium* sp.	OTU227	45	45	0	0.0283
	Pleosporales	*Sporormiella*	*Sporormiella australis*	OTU218	45	45	0	0.0085
	Pleosporales	unclassified	unclassified *Pleosporales*	OTU203	43	43	0	0.0196
Total	*Hypocreales*	*Gibberella*	*Gibberella tricincta*	OTU89	23	23	0	0.0511
	Hypocreales	*Bionectria*	*Bionectria pseudochroleuca*	OTU80	23	23	0	0.0383
	Hypocreales	*Lecanicillium*	unclassified *Lecanicillium*	OTU53	23	23	0	0.1486
	Hypocreales	*Sarocladium*	*Sarocladium kiliense*	OTU417	23	23	0	0.2487
	Chaetothyriales	*Cyphellophora*	*Cyphellophora olivacea*	OTU103	23	23	0	0.1003
	Hypocreales	*Sarocladium*	unclassified *Sarocladium*	OTU45	23	23	0	0.0507
	Eurotiales	*Penicillium*	*Penicillium sanguifluum*	OTU96	23	23	0	0.1993
	Hypocreales	*Gibberella*	*Gibberella tricincta*	OTU26	23	23	0	0.0464
	Hypocreales	*Fusarium*	*Fusarium proliferatum*	OTU400	22	22	0	0.8044
	Hypocreales	*Fusarium*	*Fusarium redolens*	OTU477	22	22	0	0.2570
