# Supplementary figures and images for: Fungal Endophytic Community and Diversity Associated with Desert Shrubs Driven by Plant Identity and Organ Differentiation in Extremely Arid Desert Ecosystem
Source: J Fungi (Basel). 2021 Jul 20;7(7):578. doi: 10.3390/jof7070578 (PMC8306007; doi:10.3390/jof7070578)

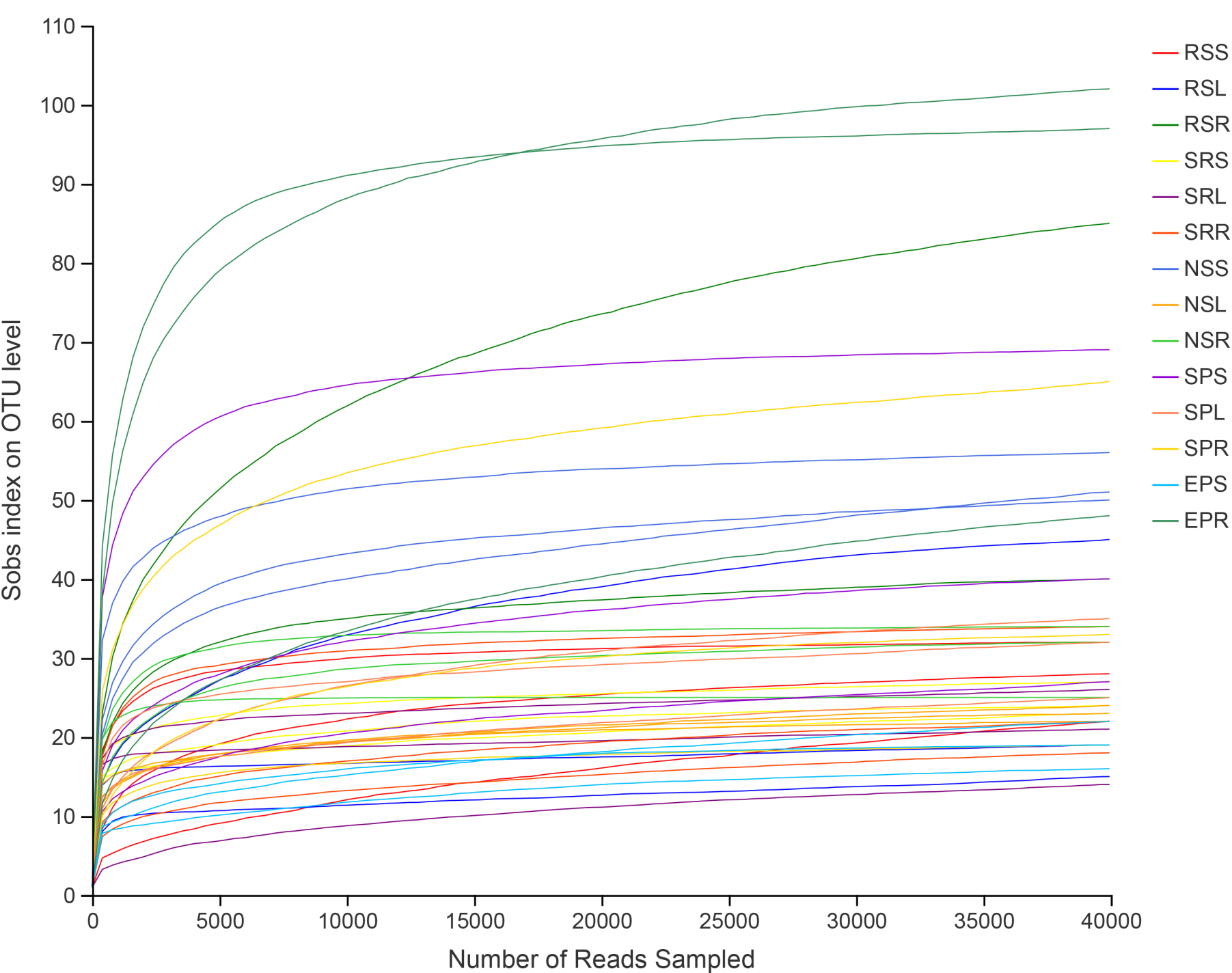

Figure S1

Supplement: Supplementary file 1 [file jof-07-00578-s001.zip › Supplementary materials/Fig.S1.pdf]

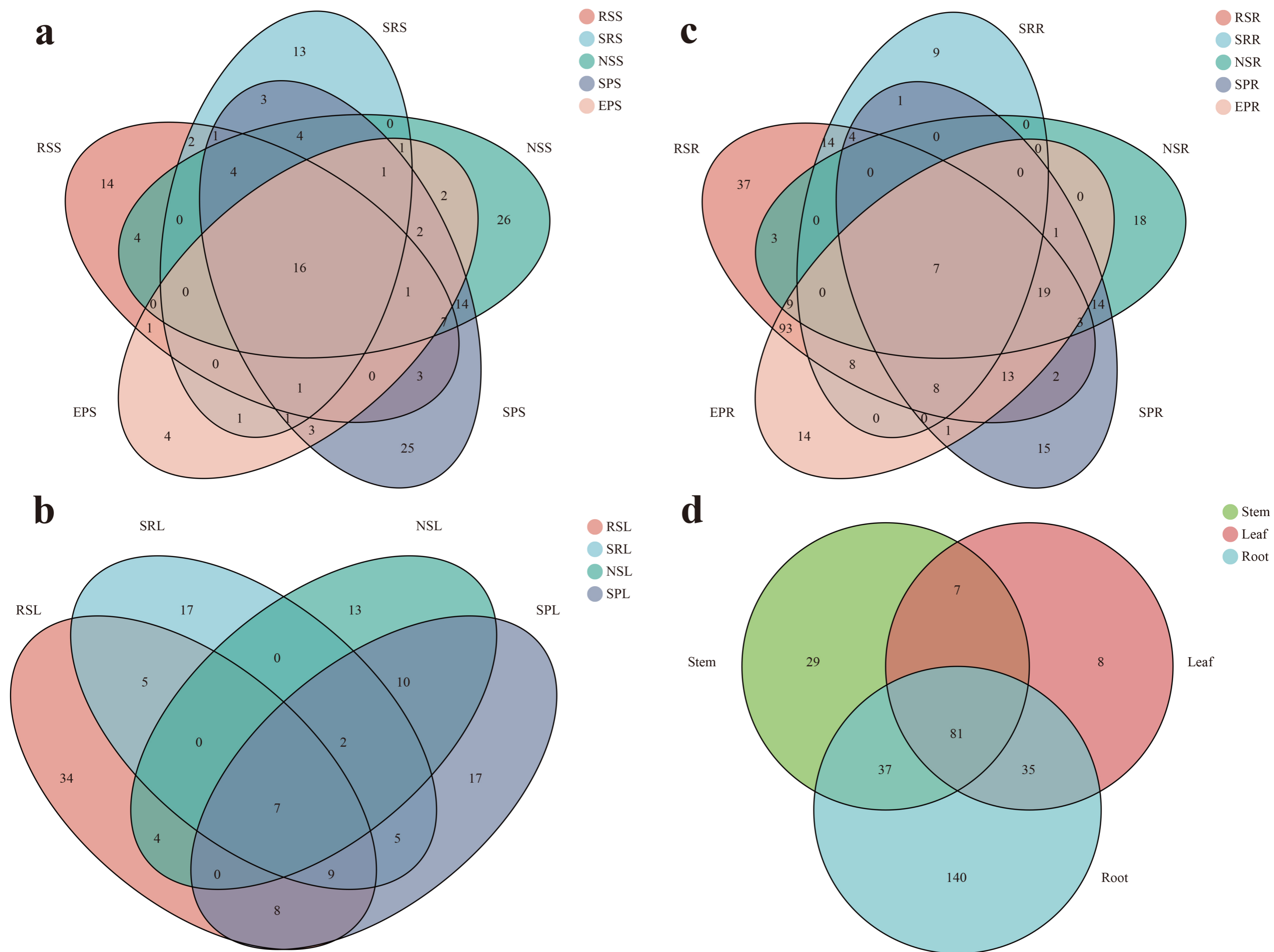

Figure S2

Supplement: Supplementary file 1 [file jof-07-00578-s001.zip › Supplementary materials/Fig.S2.pdf]

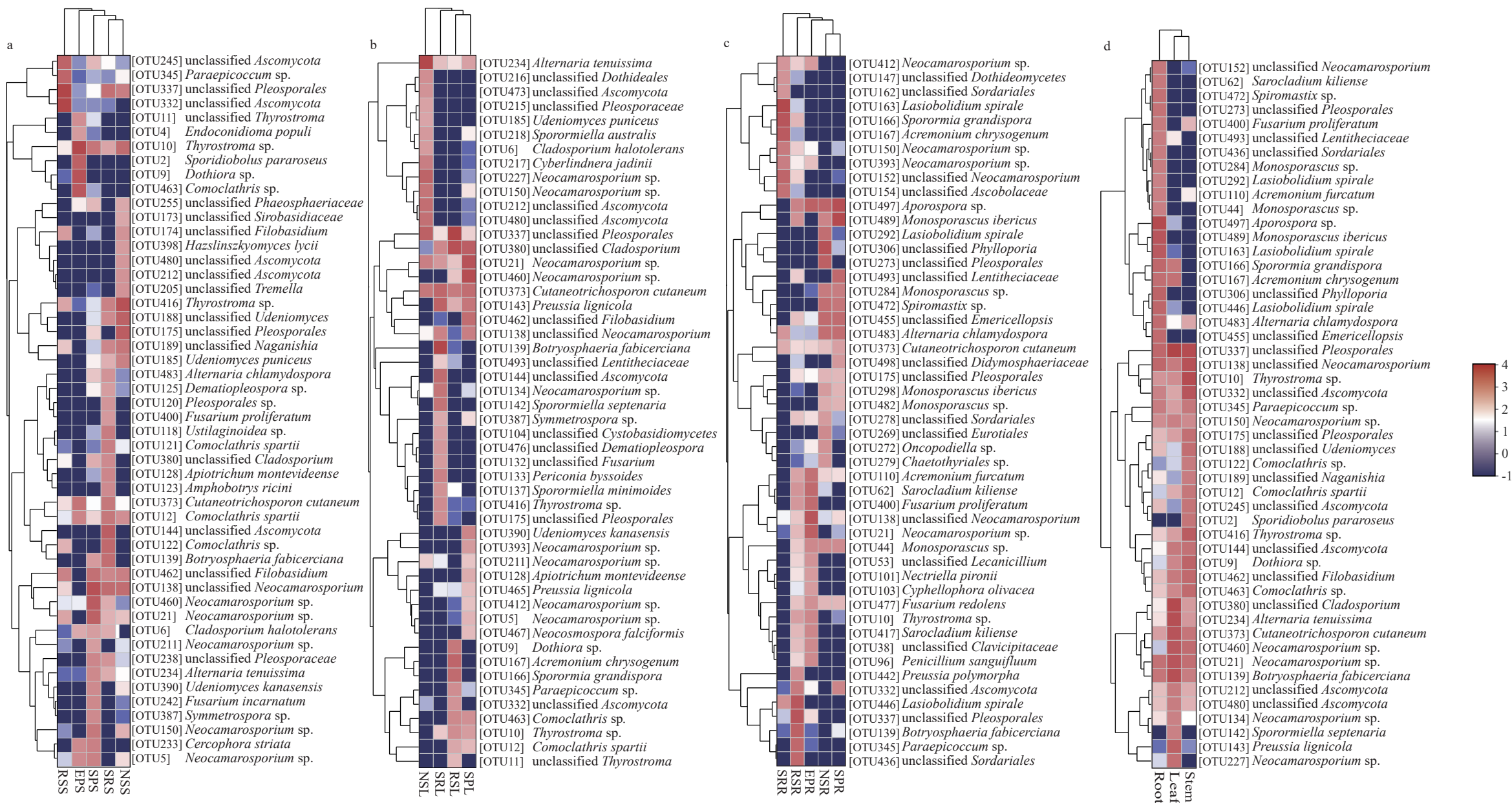

Figure S3

Supplement: Supplementary file 1 [file jof-07-00578-s001.zip › Supplementary materials/Fig.S3.pdf]
