# Supplementary material for: Fungal Endophytic Community and Diversity Associated with Desert Shrubs Driven by Plant Identity and Organ Differentiation in Extremely Arid Desert Ecosystem
Source: J Fungi (Basel). 2021 Jul 20;7(7):578. doi: 10.3390/jof7070578 (PMC8306007; doi:10.3390/jof7070578)
Supplement: Supplementary file 1 [file Fig.S4.pdf › Supplementary materials/Fig.S4.pdf]

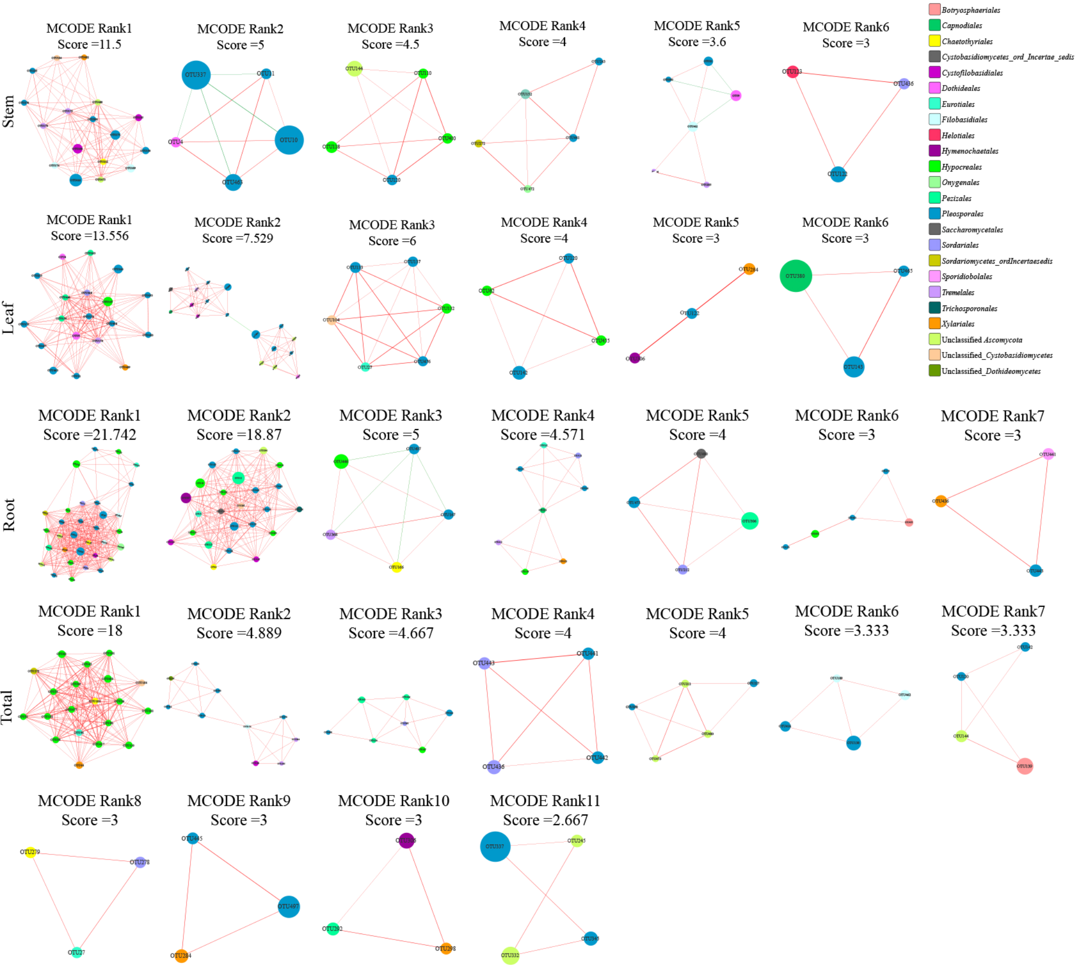
Stem
MCODE Rank1
Score =11.5
MCODE Rank2
Score =5
MCODE Rank3
Score =4.5
MCODE Rank4
Score =4
MCODE Rank5
Score =3.6
MCODE Rank6
Score =3
Leaf
MCODE Rank1
Score =13.556
MCODE Rank2
Score =7.529
MCODE Rank3
Score =6
MCODE Rank4
Score =4
MCODE Rank5
Score =3
MCODE Rank6
Score =3
Root
MCODE Rank1
Score =21.742
MCODE Rank2
Score =18.87
MCODE Rank3
Score =5
MCODE Rank4
Score =4.571
MCODE Rank5
Score =4
MCODE Rank6
Score =3
MCODE Rank7
Score =3
Total
MCODE Rank1
Score =18
MCODE Rank2
Score =4.889
MCODE Rank3
Score =4.667
MCODE Rank4
Score =4
MCODE Rank5
Score =4
MCODE Rank6
Score =3.333
MCODE Rank7
Score =3.333
MCODE Rank8
Score =3
MCODE Rank9
Score =3
MCODE Rank10
Score =3
MCODE Rank11
Score =2.667
Botryosphaeriales
Capnodiales
Chaetothyriales
Cystobasidiomycetes_ord_Incertae_sedis
Cystofilobasidiales
Dothideales
Eurotiales
Filobasidiales
Helotiales
Hymenochaetales
Hypocreales
Onygenales
Pezizales
Pleosporales
Saccharomycetales
Sordariales
Sordariomycetes_ordIncertaesedis
Sporidiobolales
Tremelales
Trichosporonales
Xylariales
Unclassified Ascomycota
Unclassified_Cystobasidiomycetes
Unclassified_Dothideomycetes

Figure S4
